# The Effects of Sishen Wan on T Cell Responses in Mice Models of Ulcerative Colitis Induced by Dextran Sodium Sulfate

**DOI:** 10.1155/2021/9957709

**Published:** 2021-12-16

**Authors:** Ke Li, Jiamin Dong, Dongyu Ge, Mengjia Li, Hehe Ye, Xudan Wang, Ying Wu

**Affiliations:** ^1^School of Life Sciences, Beijing University of Chinese Medicine, Beijing 102400, China; ^2^Liuzhou Key Laboratory for Infectious Disease Immunology Research, Guangxi Health Commission Key Laboratory for Clinical Biotechnology, Liuzhou People's Hospital Affiliated to Guangxi Medical University, Liuzhou 545006, China

## Abstract

Currently, it is unclear whether Sishen Wan (SSW) could modulate the balance of Th1 cells, Th17 cells, and Tregs and we evaluated the effects of SSW on T cell responses in mice models of ulcerative colitis (UC). The mice models of acute UC (4% dextran sodium sulfate (DSS), 8 days) and chronic UC (3% DSS, 16 days) with SSW were assayed. Colon tissues were collected for immunohistochemical analysis, enzyme linked immunosorbent assay (ELISA), and flow cytometry (FCM). The expressions of cytokines associated with Tregs, transcription factors of Th17 cells, the frequencies of Th1 cells, Th17 cells, and Tregs, and the functional plasticity of Th17 cells were detected. The frequency of IFN-*γ*^+^ T cells was not changed significantly with SSW treatment in acute DSS. In chronic models, the frequency of IFN-*γ*^+^ T cells was downregulated with SSW. Meanwhile, the levels of ROR*γ*t and the frequency of IL-17A^+^ Th17 cells showed no significant differences after SSW treatment. Despite no significant effect on the transdifferentiation of Th17 cells in chronic UC models, SSW transdifferentiated Th17 cells into IL-10^+^ Th17 cells and downregulated IFN-*γ*^+^ Th17 cells/IL-10^+^ Th17 cells in acute DSS. Moreover, there were no significant changes of cytokines secreted by Tregs in acute DSS after SSW treatment, but SSW facilitated the expressions of IL-10 and IL-35, as well as development of IL-10^+^ Tregs in chronic DSS. SSW showed depressive effects on the immunoreaction of Th17 cells and might promote the conversion of Th17 cells into IL-10^+^ Th17 cells in acute UC, while it inhibited the excessive reaction of Th1 cells, facilitated the development of Tregs, and enhanced the anti-inflammatory effects in chronic UC.

## 1. Introduction

Ulcerative colitis (UC), a common chronic nonspecific inflammatory bowel disease (IBD), is generally manifested as diarrhea, abdominal pain, weight loss, and hematochezia [1, 2]. Currently, the prevalence of UC is increasing worldwide. It is commonly recognized that extensive and long-standing UC poses a substantial cancer risk and the risk for colitis-associated colorectal cancer (CAC) was reported to be around 30% at 35 years after the onset of the disease [3, 4]. In China, UC prevalence increased 3 times from 2001 to 2011 and the incidence of UC was approximately 1.18/100,000 in 2017 [5, 6]. In USA, UC prevalence rates in 2007 and 2017 were 0.25% and 0.39%, respectively [7]. Current therapeutic options used for the treatment against UC include 5-aminosalicylate, glucocorticoid, and immunosuppressive agents. However, these available treatments are accompanied with significant adverse gastrointestinal reactions, serious complications such as *tuberculosis*, recurrence, and a heavy economic burden [8–10]. It is of vital importance to explore a series of novel, safe, and efficient methods for the treatment of UC.

Although the etiology and pathogenesis of UC remain elusive, it is generally accepted that UC is associated with disordered immune response, genetic susceptibility, intestinal microbiota disorder, and environmental factors [11]. T helper 1 (Th1) cells, T helper 17 (Th17) cells, and regulatory T cells (Tregs) have been found to play important roles in the pathogenesis of UC. Th1 cells, which produce and secret proinflammatory cytokines such as Interferon-gamma (IFN-*γ*), Interleukin-2 (IL-2), and tumor necrosis factor-alpha (TNF-*α*), are known to play a vital role in cellular immunity [12]. With the upregulation of T-box transcription factor expressed in T-cells (T-bets), Th1 cells are induced excessively by signal transducer and activator of transcription 4 (STAT4) and STAT1 signal pathways, which are activated by IL-12 derived from dendritic cells and IFN-*γ* produced from natural killer (NK) cells [13–18]. Th1 cells secrete IFN-*γ* and exacerbate inflammatory injury [19]. Th17 cells are associated with the defense against extracellular infectious factors, inflammatory responses, and the development of autoimmunity [20, 21]. After the increased expression of retinoic acid-related orphan receptor gamma *t* (ROR*γ*t), the polarization of Th17 cells is mediated by overexpressed IL-1*β* and IL-6 and activated STAT3 in UC patients [22]. Th17 cells produce and secrete IL-17 and IFN-*γ*, which induce epithelial cells to release granulocyte colony-stimulating factor (G-CSF), accelerate neutrophil recruitment, and mediate excessively inflammatory reactions [15, 23]. However, IL-22, another effector cytokine derived from Th17 cells, facilitates epithelial cells to secrete antimicrobial peptides, augment epithelial repair, and restore barrier function [23, 24]. With remarkable functional plasticity, IL-17A producing Th17 cells readily acquire the ability to secrete IFN-*γ* in UC model, while limiting excessive innate effector functions of dendritic cells (DCs) and macrophages by expressing IL-10. Expressing anti-inflammatory cytokines such as IL-35, transforming growth factor (TGF-*β*) [25–27], and IL-10, Foxp3^+^ Tregs are widely distributed in mucosal tissue of colon and depress excessive inflammatory responses [28–30]. In UC patients, the expression of IL-10 is downregulated with the impaired Foxp3^+^ Tregs and the immunologic injury is aggravated [31].

As a source of pharmaceutical material, Chinese medicine plays an important role in the therapy against UC. Sishen Wan (SSW), described in *Chen shi xiao er dou zhen fang lun*, written by Chen, Wenzhong in Song Dynasty, is composed of *Myristica fragrans* Houtt. (200 g), *Psoralea corylifolia* L. (400 g), *Schisandra chinensis* Turcz. (Baill.) (200 g), *Euodia rutaecarpa* (Juss.) Benth. (100 g), and *Ziziphus jujube* Mill. (200 g) [32] (Table 1).

SSW is applied to treat diarrhea of deficiency of spleen-yang (the heating effect of digesting food) and kidney-yang (the source power of human life) via the efficacies of warming kidney (strengthening kidney-yang) for dispelling cold, warming yang to resolve wetness (treating a series of diseases with the syndromes of heavy sensation of head, dizziness, and aches in the limbs), and relieving diarrhea with astringents in the theory of Chinese medicine. SSW has shown beneficial effects on UC in clinic treatments and several clinical studies have demonstrated that SSW can ameliorate abdominal pain, relieve diarrhea, and alleviate intestinal mucosal hyperemia and hematochezia in UC patients [33–35]. Sun et al. reported that the appetite of UC patients was improved by salicylazosulfapyridine (SASP) combined with SSW, and SASP combined with SSW effectively relieved abdominal pain and diarrhea [36]. The clinically effective rate of SSW on 36 UC patients was 94.4% reported by Hu and was 92.31% on 26 UC patients described by Wang [37, 38]. Modern pharmacodynamic and pharmacological studies also confirmed that SSW was effective. In dextran sodium sulfate- (DSS-) induced experiment on mouse models of colitis, SSW attenuated histopathological injuries, recovered colonic mucosa gradually, restored colonic length, downregulated contents of IL-2, IL-7, IL-12, and IL-15, upregulated IL-10 expression, regulated the quantity and subpopulation of central memory T (Tcm) and effector memory T (Tem) cells, suppressed the activation of phosphatidylinositol 3-kinase (PI3K), protein kinase B (Akt), and T-box protein expressed in T cells (T-bet) [39]. In DSS-induced UC mice, SSW downregulated the levels of IL-1*β*, IL-4, IL-9, and IL-17A and CD11C^+^CD103^+^E-cadherin^+^ cells, as well as increasing the beneficial bacteria and reducing the pathogenic bacteria [8]. Wang et al. reported that the expressions of nuclear factor kappa B (NF-*κ*B) p65 and NF-*κ*B essential modulator- (NEMO-) like kinase (NLK) were depressed and the transcriptions of IFN-*γ*, IL-12/23, TNF-*α*, IL-17, and IL-1*β* genes were inhibited in SSW-treated UC rats [40]. After the therapy of SSW, inflammatory damage and congestive and edema were alleviated, the infiltration of inflammatory cells was ameliorated, the recoveries of epithelial damage and ulceration were accelerated, and the expression of IL-10 was upregulated in dextran sodium sulfate- (DSS-) induced UC mice [41]. In our previous studies, SSW ameliorated the infiltration of inflammatory cells in colonic tissues in TNBS and DSS-induced mice models and improved disease activity index (DAI) [42]. Moreover, SSW could alleviate inflammatory damage by depressing the activity of myeloperoxidase (MPO) in acute and chronic UC, treat acute UC by reducing the contents of IFN-*γ* and IL-17A, and relieve the damage in chronic UC by accelerating the secretion of IL-22 [43, 44]. Our study revealed that SSW was effective in ameliorating UC via T cell responses, and we discussed the different therapeutic mechanisms of SSW on T cells in acute and chronic UC mice.

## 2. Material and Methods

### 2.1. Chemicals and Reagents

DSS (molecular weight: 6500–10000, No. Lot#SLBB4215V) and Concanavalin A (ConA, No. 71K7024) were purchased from Sigma-Aldrich. Collagenase IV was obtained from Gibco, New York, USA (250 units/mg, No. 1371044). Cell Stimulation Cocktail (containing PMA and ionomycin, No. E13494-112), Intracellular Fixation and Permeabilization Buffer (No. E09680-1642), Anti-Mouse CD4 antigen presenting cell (APC, No. E07036-1634), Anti-Mouse IFN gamma PE (No. E02134-1631), Anti-Mouse IL-17-FITC (No. E00850-1631), Anti-Mouse ROR*γ*t mAb (No. E05251-1630), Anti-Mouse Foxp3 mAb, and Anti-Mouse IL-10 PE (No. E02094-1633) were acquired from EBioscience, California, USA. Percoll was purchased from Pharmacia, Uppsala, Sweden, and Dithiothreitol (DTT) was acquired from Promega, Madison, USA. DNase was obtained from Biodee, Beijing, China (No. R0110). PV-9001 Polink-2 plus® Polymer HRP Detection System including 3% H_2_O_2_, Reagent 1(Polymer Helper) and Reagent 2 (HRP-labeled Goat Anti-Rabbit IgG), HRP-labeled Goat Anti-Mouse IgG, and Mouse anti-*β*-Actin Monoclonal Antibody were purchased from Beijing Zhongshan Jinqiao Biotechnology Co., Ltd., Beijing, China.

### 2.2. Animals and Drugs

All animal treatments and experiments were overseen and approved by experimental animal ethics subcommittee of academic committee in Beijing University of Chinese Medicine and were supported by a grant from Beijing University CM (Ethics approval number: BUCM-4-2020090101-3001). Animal studies were carried out on 60 specific pathogen-free (SPF) female C57BL/6J mice (Beijing Huafukang Biotechnology Co., Ltd., Beijing, China, license No: SCXK 2009–0007), that were 8 to 10 weeks old, weighing 18–22 g. SSW was purchased from Beijing Tongrentang Natural Medicine Co., Ltd. (No. 13080010).

### 2.3. Experimental Design

After adaptation for 7 days, mice were randomly divided into six experimental series, with each group consisting of 10 mice: acute control, chronic control, acute DSS, acute DSS + SSW, chronic DSS, and chronic DSS + SSW. Mice in acute control were not challenged with DSS and treated with SSW for 5 days. Mice in chronic control were not induced by DSS and treated with SSW for 26 days. Models in acute DSS were induced through free access to 4% (*w*/*v*) DSS dissolved in distilled water for 5 days. Mice in acute DSS + SSW were given SSW by gavage from day 2 to day 9 after the start of experiment, once a day, and the dose of SSW was calculated by the following equation:

(1) $$\begin{array}{c} D_{m}=D_{h}\times \frac{F}{W}, \end{array}$$

where *D*_*h*_ is the clinical dose of SSW for adult humans, *D*_*m*_ is the administered dose of SSW for mice, *W* is the weight of the adult human body, and *F* is the dose conversion factor for mice and adult humans [45]. The clinical dose of SSW is 9–18 g per day [32], and we chose the average of 9 g and 18 g (13.5 g). *W* was set as 60 kg. Considering the dose conversion factor of 10 between mouse and human, *D*_*m*_ of 2.25 g/kg was calculated in our study [45].

Mice were sacrificed by cervical dislocation. Colon tissues were collected quickly and aseptically. Mice in chronic DSS were established by drinking 3% (*w*/*v*) DSS on day 1 to day 5, day 8 to day 12, day 15 to day 19, and day 22 to day 26, respectively, and drinking water without DSS on day 6 to day 7, day 13 to day 14, and day 20 to day 21 [46–48]. Mice in chronic DSS + SSW were intragastrically administered by SSW on day 12 for 16 days and were euthanized by cervical dislocation on day 29. Colon tissues were harvested aseptically.

### 2.4. Immunohistochemistry Assay

Colon tissues (1 cm) were fixed in 10% formalin, embedded in paraffin, and cut into 4 *μ*m thick sections. The sections were dewaxed, hydrated, and incubated with 3% H_2_O_2_ for 5–10 min block endogenous peroxidase activity and washed with phosphate buffer saline (PBS) 3 times, 2 min each time. The tissues were processed with antibody against ROR*γ*t, incubated at 37°C for 1–2 h, and washed by PBS 3 times, 2 min each time. Reagent 1 was added into tissues and tissues were incubated at 37°C for 10–20 min and washed by PBS 3 times. Then, tissues were incubated with Reagent 2 at 37°C for 10–20 min and washed by PBS. Sections were colored by using 3, 3′-Diaminobenzidine tetrahydrochloride hydrate (DAB) and counterstained with hematoxylin. The samples were observed under Nikon ECLIPSE TI-U inverted microscope (Nikon, Tokyo, Japan) and images were captured. Using Image Pro Plus 6.0 system, the mean absorbance of ROR*γ*t^+^ area was measured.

### 2.5. Enzyme Linked Immunosorbent Assay (ELISA)

ELISA was accessed to detect the expressions of IL-10, TGF-*β*, and IL-35 in colon tissues. Colon tissues were cultured in 24-well plates with RPMI 1640 medium. Colon tissues were incubated at 37°C in a 5% CO_2_ atmosphere for 24 h and culture supernatant was collected. Immunoreactive IL-10, TGF-*β*, and IL-35 in colon tissues were evaluated quantitatively by ELISA as per the manufacturer's instructions.

### 2.6. Cell Isolation

The colon tissues were rinsed with Hank's buffer, incised longitudinally, and cut into pieces. The pieces were incubated with 0.37 mg/ml EDTA and 0.145 mg/ml DTT in Hank's buffer in 37°C water bath 2 times, 20 min each time, and the suspension, mainly containing epithelial cells and intraepithelial lymphocytes, was removed. The remaining tissues were supplemented with 25 ml RPMI 1640 digestive solution containing 5% fetal bovine serum (FBS), 0.5 mg/ml collagenase, and 0.1 mg/ml DNase and incubated at 37°C water bath 3 times, 30 min each time. The supernatant was collected and filtered with 1 mm strainer and 100 *μ*m and 50 *μ*m polyamides and separated single cells were acquired. Cells were washed by RPMI-1640 containing 5% FBS and the survival rate was measured by trypan blue. Then, cells were filtered by abacterial absorbent cotton. Discontinuous gradient centrifugation was accessed by 40% and 100% Percoll to enrich the ratio of lamina propria mononuclear cells (LPMCs) in a white ring at the interphase of 40% Percoll and 70% Percoll solutions.

### 2.7. Flow Cytometry (FCM)

LPMCs were stained with fluorochrome-conjugated antibodies directed CD4 against cell surface expression on APC for 30 min in the dark and rinsed twice by centrifuging with 400 ×g for 5 min at 4°C with Fluorescence Activated Cell Sorting (FACS) buffer. Samples were fixed with an equal volume of Intracellular Fixation buffer for 20 min on a rotating platform in the dark. Then, cells were mixed with Permeabilization buffer and centrifuged at 300–400 ×g for 5 min and the supernatant was removed. Samples were resuspended with 100 *µ*l of 1x Permeabilization buffer, added with 10 *μ*l of 0.025 mg/ml anti-IL-10-PE, FITC-IL-17A, or PE-IFN-*γ*, and incubated for 20 min in the dark. Then, cells were rinsed by FACS buffer twice and resuspended with 0.5 ml FACS buffer. Finally, the percentages of IL-10^+^ CD4^+^ T cells, IL-17A^+^ CD4^+^ T cells, and IFN-*γ*^+^ CD4^+^ T cells in LPMCs were detected by FCM. For detecting the functional plasticity of Th17 cells, the samples were added with Anti-Mouse/Rat IL-17A FITC and Anti-Mouse IFN-*γ* PE or Anti-Mouse/Rat IL-17A FITC and Anti-Mouse IL-10 PE and analyzed by FCM.

### 2.8. Statistical Analysis

The data were presented as mean ± standard deviation. Statistical analysis was carried out using SPSS 16.0. A one-way analysis of variance (ANOVA) with LSD test and Tamhane's T2 test was performed to determine statistical differences. *P* < 0.05 was considered statistically significant.

## 3. Results

### 3.1. SSW Depressed the Expression of Th17 Cells in Acute UC Mice Models and Inhibited Th1 Cells in Chronic UC Models

Predominant pathogenic Th1 cell immune responses were considered to link to the pathogenesis of UC in classical view. However, IL-17 signaling pathway derived from Th17 cells has been discovered as a distinctive signal pathway associated with the pathogenesis of UC [49]. Our previous studies elaborated that SSW might alleviate immunologic injuries by downregulating the levels of IFN-*γ* and IL-17A in acute DSS and relieve the damage in chronic DSS by promoting the secretion of IL-22. This study revealed the frequencies of Th1 and Th17 cells and the expression of ROR*γ*t in SSW-treated UC mice.

**Figure 1:**
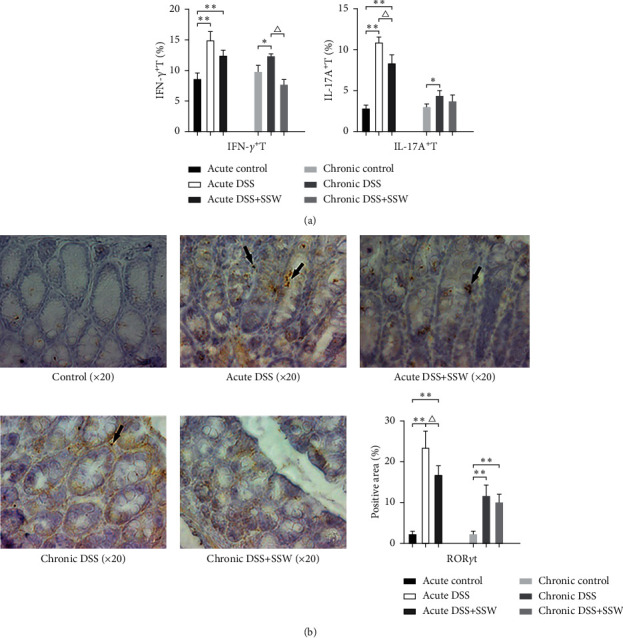
The effects of SSW on the responses of effective T cells in acute DSS on day 9 and chronic DSS on day 28 (*n* = 10 per group). (a) The proportions of IFN-*γ*^+^ T cells and IL-17A^+^ T cells in LPMC derived from colonic tissues in acute and chronic DSS were detected by cytometry (%). (b) Expression of ROR*γ*t in colonic tissues was detected by immunohistochemical method. The ROR*γ*t^+^ areas were marked by black arrows. ^*∗*^*P* < 0.05 and  ^*∗∗*^*P* < 0.01 versus acute or chronic control. ^△^*P* < 0.05 and ^△△^*P* < 0.01 versus acute or chronic DSS.

FCM analyses of IFN-*γ*^+^ T cells and IL-17A^+^ T cells are shown in Figure 1(a). Significant upregulation in the percentages of IFN-*γ*^+^ T cells and IL-17A^+^ T cells were observed in acute DSS and chronic DSS (*P* < 0.01). The percentages of IL-17A^+^ T cells were significantly lower with SSW in acute DSS, whereas there was no significant tendency of downregulation in the percentage of Th1 cells. In chronic DSS, SSW decreased the percentages of IFN-*γ*^+^ T cells and IL-17A^+^ T cells.

The results showed that ROR*γ*t expression was increased significantly (*P* < 0.01) in acute and chronic DSS compared with control mice. With the therapy of SSW, the level of ROR*γ*t was decreased in acute DSS and showed no significant change in chronic DSS (Figure 1(b)). From this result, it could be seen that the effect of SSW on the level of ROR*γ*t was consistent with IL-17A.

### 3.2. SSW Modulated the Plasticity of Th17 Cells in Acute UC Mice

**Figure 2:**
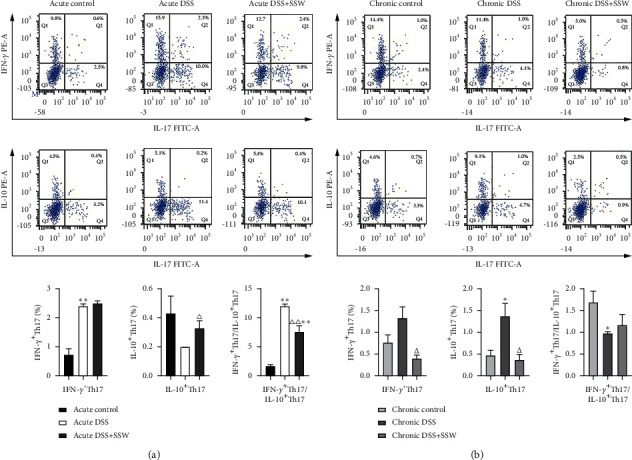
Plasticity of Th17 cells from LPMC of mice induced by DSS (*n* = 10 per group). (a) The proportions of IFN-*γ*^+^ Th17 and IL-10^+^ Th17 cells from LPMC derived from colonic tissues in acute DSS were detected by FCM (%) on day 9. (b) The proportions of IFN-*γ*^+^ Th17 and IL-10^+^ Th17 cells from LPMC in chronic DSS were detected by FCM (%) on day 28. ^*∗*^*P* < 0.05 and  ^*∗∗*^*P* < 0.01 versus acute or chronic control. ^△^*P* < 0.05 and ^△△^*P* < 0.01 versus acute or chronic DSS.

A low frequency of Th17 cells in LPMC is capable of expressing nonspecific cytokines such as IFN-*γ*, a proinflammatory cytokine, or IL-10, an anti-inflammatory cytokine. FCM was used to detect the plasticity of Th17 cells in acute and chronic DSS treated with SSW. As presented in Figure 2(a) (*P* < 0.05), the percentage of IFN-*γ*^+^ Th17 cells was significantly increased, while the frequency of IL-10^+^ Th17 cells was decreased with no statistical significance in acute DSS, leading to the upregulation of IFN-*γ*^+^ Th17 cells/IL-10^+^ Th17 cells. SSW had no significant effect on IFN-*γ*^+^ Th17 cells and increased the frequency of IL-10^+^ Th17 cells, which downregulated the IFN-*γ*^+^ Th17 cells/IL-10^+^ Th17 cells. Our results indicated that SSW might promote the conversion to IL-10^+^ Th17 cells in acute DSS.

The frequency of IL-10^+^ Th17 cells was increased significantly and there was no significant upregulation of IFN-*γ*^+^ Th17 cells in mice model of chronic colitis, resulting in the decrease of IFN-*γ*^+^ Th17 cells/IL-10^+^ Th17 cells. The levels of IFN-*γ*^+^ Th17 cells and IL-10^+^ Th17 cells were reduced with SSW and there was no significant change in IFN-*γ*^+^ Th17 cells/IL-10^+^ Th17 cells. Thus, the therapeutic mechanisms of SSW might be independent of IFN-*γ*^+^ Th17 cells/IL-10^+^ Th17 cells in chronic DSS (Figure 2(b), *P* < 0.05).

### 3.3. SSW Promoted Tregs to Secrete Inhibitory Cytokines in Chronic UC Models

**Figure 3:**
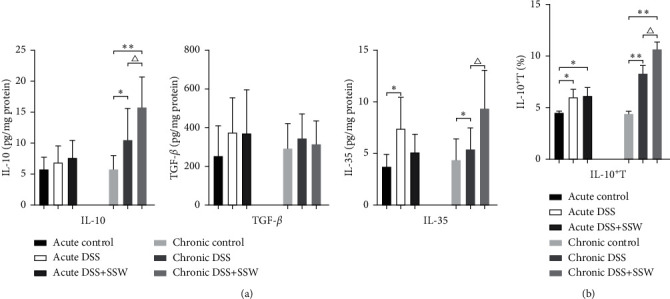
The effects of SSW on the responses of Tregs in acute DSS on day 9 and chronic DSS on day 28 (*n* = 10 per group). (a) Levels of IL-10, TGF-*β*, and IL-35 from the supernatant from culture of colonic tissues in acute and chronic DSS were measured by ELISA. (b) The proportions of IL-10^+^ T cells in LPMC derived from colonic tissues in acute and chronic DSS were detected by cytometry (%). ^*∗*^*P* < 0.05 and  ^*∗∗*^*P* < 0.01 versus acute or chronic control. ^△^*P* < 0.05 and ^△△^*P* < 0.01 versus acute or chronic DSS.

The level of IL-35 was increased obviously in acute DSS when compared with healthy mice; however, there were no significant differences of IL-10 and TGF-*β* between mice in acute control and acute DSS. Our results suggested that the levels of some inhibitory cytokines were increased in acute DSS. There were no significant effects on the contents of IL-10, TGF-*β*, and IL-35 in acute DSS treated with SSW, which might be due to the fact that the efficacy of SSW had no obvious correlation with inhibitory cytokines derived from Tregs. The level of IL-10 was upregulated significantly in chronic UC models, while the levels of TGF-*β* and IL-35 had no obvious variations. SSW further increased the levels of IL-10 and IL-35, which implied that SSW might repress excessive inflammatory responses by upregulating the contents of IL-10 and IL-35 (Figure 3(a), *P* < 0.05).

Significant upregulation in the percentage of IL-10^+^ T cells was found in acute DSS and chronic DSS. SSW further increased the frequency of IL-10^+^ T cells in chronic DSS; however, this effect was not obvious in acute DSS (Figure 3(b), *P* < 0.05). Our results indicated that SSW might inhibit excessive inflammatory responses and facilitate tissue reconstruction by enhancing the frequency of IL-10^+^ T cells in chronic UC.

## 4. Discussion

With alteration of epithelial structure, high-level neutrophils and lymphocytes infiltration, reduction of colon length, destruction of mucosa, and loss of crypts, a series of symptoms such as significant weight loss, rectal bleeding, diarrhea, and deteriorated inflammation are evident in DSS-induced animal models [50, 51]. The mechanism of mucosal injury induced by DSS has not been completely explicit and may be associated with the interaction between DSS and epithelial barrier [52, 53]. Intestinal epithelial bacteria and bacterial metabolites are translocated, caused by the damage to the epithelial barrier induced by DSS, leading to a large quantity of lipopolysaccharides (LPS) entering the cytoplasm, resulting in downregulation of Thioredoxin-interacting protein (TXNIP), excessively upregulating the expressions of IL-1*β* and IL-18, and causing intemperate inflammatory responses [54–56]. Furthermore, DSS accelerates the differentiation to Th17 cells, increasing the expressions of IL-17 and ROR*γ*t, while inhibiting the polarization to Tregs, leading to inflammatory injuries and alterations of immunocytes and cytokines [57–60]. Therefore, DSS-induced mice models were used to explore the effects and immunologic mechanisms of SSW against UC. As a famous traditional Chinese herbal medicine, SSW was applied to treat diarrhea caused by deficiency of spleen-yang and kidney-yang (the source power of human life) in the theory of Chinese medicine. SSW is frequently used in clinical therapies of ulcerative colitis, irritable bowel syndrome, and allergic colitis. The clinical benefit rate in clinical trial of SSW combined with enema of herbal medicine on 62 cases of chronic UC was 90%, as reported by Yang and Rongfeng [61]. As described by Xie and Jianjun, the clinically effective rate of SSW combined with enema on 58 cases of UC was 89.7% [62]. Hu reported that the clinical benefit rate of SSW on 36 cases of UC was 94.4% [37]. Results from experimental animals revealed that SSW and major bioactive components of SSW might alleviate chronic colitis by depressing Wnt/*β*-catenin pathway [63]. Accomplished by decreasing the levels of MPO and malondialdehyde (MDA), increasing the contents of superoxide dismutase (SOD), IL-4, and IL-10 mRNA expressions, SSW might have a protective effect on impaired colonic mucosa rats with UC [64]. Moreover, SSW might decrease the expressions of IFN-*γ*, IL-1*β*, and IL-17, upregulate the level of IL-4, and show therapeutic effects in chronic colitis [40]. Our preliminary studies revealed that SSW reduced DAI significantly, attenuated infiltration of inflammatory cells, decreased the activity of MPO in colonic tissues, and ameliorated inflammatory damages in UC mice models [43, 65]. This study exhibited the immunologic regulation of SSW on T cell responses in acute and chronic UC mice models.

The dysregulation of Th cells induced by DSS is crucial in the damage of colon tissues during the acute and chronic stages of UC. T-bet signaling pathway is induced by the activation of STAT1 in IBD process and facilitates the differentiation to Th1 cells, and Th1 cells produce and secrete IFN-*γ* and IL-2, which repress the differentiation to Th2 cells and restrain the expressions of IL-5 and IL-13 derived from Th2 cells, leading to the disequilibrium of Th1/Th2; in addition, IFN-*γ* promotes macrophages to secrete large amounts of proinflammatory cytokines and exacerbates the tissue damage [58, 66–69]. Our previous investigation revealed that the therapeutic effects of SSW were mediated by depressing the levels of IFN-*γ* and IL-17A in acute UC mice models and upregulating the content of IL-22 in chronic colitis [44]. In this study, FCM showed that SSW significantly restrained the frequency of IFN-*γ*^+^ T cells in chronic DSS; however, this effect was not obvious in acute DSS, which might be due to the fact that the efficacy of SSW was not associated with Th1 cells. Under normal conditions, Th17 cells, broadly distributed on intestinal epithelial tissues, secrete cytokines such as IL-17A, IL-17F, IL-22, and IL-21 and proceed with moderate immune responses in allusion to intestinal flora to maintain the stabilization of colon mucosa; in the pathological process of UC, IL-6, IL-23, and IL-21 are produced and secreted by activated macrophages, epithelial cells, and dendritic cells (DCs), followed by facilitating the expression of ROR*γ*t, a critical transcription factor for Th17 cells differentiation, resulting in the differentiation to Th17 cells [23, 70–73]. Immunologic responses, mediated by IL-17A, make a protective effect on immune balance by eliminating pathogen; however, excessive IL-17A causes the imbalance between Th17 cells/Tregs and is strongly linked to the pathogenesis of UC [74–76]. Our results further showed that ROR*γ*t and IL-17A^+^ T cells were upregulated in acute DSS and downregulated after being treated with SSW; however, these variations were not significant in chronic UC mice. During the acute stage of UC, the neutrophil recruitment to the colon is promoted by Th17 immune responses and contributes to the exacerbation of UC activities [77]. Thus, SSW might suppress the neutrophil recruitment to the colon by inhibiting Th17-mediated immunologic reactions in acute DSS. Mechanistically, fibrosis is a consequence of chronic stage of UC [78]. In the chronic DSS, the fibrogenesis in murine colitis might be ameliorated with the treatment of SSW by suppressing the production of Th1 type cytokines and polarizing macrophages towards the alternatively activated macrophages (M2 macrophages) [79].

By possessing the ability to repolarize towards different phenotypes, Th17 cells are capable of expressing IFN-*γ*, a distinctive cytokine of Th1 cells, and IL-10, a characteristic cytokine of Tregs, which is commonly referred to as plasticity [80]. Induced by the activity of proinflammatory cytokines such as IL-12 or TNF-*α*, Th17 cells express T-bet and transdifferentiate to Th1-like Th17 cells, which secrete IFN-*γ* and GM-CSF [81]. Th1-like Th17 cells participate in the pathogenesis of autoimmune diseases actively. In juvenile idiopathic arthritis patients, Th17 cells transdifferentiated to Th1-like Th17 cells after being exposed to TNF-*α* and produced IFN-*γ* and TNF-*α*, which exacerbated inflammation, as described by Maggi et al. [82]. Stimulated by anti-CD3 mAb, Th17 cells shift towards IL-10^+^ Th17 cells and produce IL-10 to play a role in immunosuppression [26, 83]. Our data suggested that Th17 cells participated in inflammatory responses by the coexpression of IFN-*γ* in acute UC, while SSW upregulated the frequency of IL-10^+^ Th17 cells. In chronic UC models, the frequency of IFN-*γ*^+^ Th17 cells was decreased compared with acute UC, which might be because upregulated IFN-*γ*^+^ Th17 cells/IL-10^+^ Th17 cells facilitated the secretion of IL-10 followed by depressing inflammation and accelerating the tissue repair in the disease process of chronic UC models. Moreover, we observed that the therapeutic mechanisms of SSW did not correlate with IFN-*γ*^+^ Th17 cells/IL-10^+^ Th17 cells in chronic UC. The possible reason for the inconspicuous effects with SSW on the variation of IFN-*γ*^+^ Th17 cells/IL-10^+^ Th17 cells in chronic UC might be the decline of Th17 cells, and potential immunologic mechanism of the therapy against chronic UC with SSW needed to be further studied.

Widely distributed in intestinal mucosal tissues, Tregs mediate immunologic tolerance to dietary antigens and gut microbiota, secrete inhibitory cytokines to ameliorate inflammatory injuries, and accelerate the reconstruction of tissues [84]. A previous study indicated that Foxp3^+^ Tregs were downregulated in peripheral blood of UC patients and upregulated in the intestinal mucosa, which depressed effector T cells in mucosal tissues [85]. Secreted by Foxp3^+^ Tregs, IL-10, TGF-*β*1, and IL-35 alleviate excessive immunologic reactions and improve UC symptoms by activating Smad signal pathway, depressing Th1 cells and Th17 cells [86–88]. We detected the variations of IL-10, TGF-*β*1, and IL-35 in intestinal mucosa and found that IL-10, TGF-*β*1, and IL-35 were increased in acute UC; however, these factors had no obvious changes with the supplement of SSW. Our results implied that the preventive and therapeutic effects of SSW might not correlate with the regulation on Tregs in acute UC. In chronic UC models, IL-35 and IL-10 were further upward with SSW. Previous studies confirmed that Th2 cells play an important role during the chronic stage of UC [89] and Th2 cells also secrete IL-10 [90]. Th2 cells depress the excessive inflammatory responses in UC; however, Th2 polarized immune responses were associated with fibrosis development [91]. Treg-derived IL-35 reduces the excessive Th2 immune responses [92]. Thus, we speculated that SSW might promote the IL-10 expression by Tregs and Th2 cells to inhibit inordinate immune responses and facilitate the expression of IL-35 to suppressive excessive Th2 immune responses in chronic UC rather than acute UC. However, the mechanisms of SSW on Th2 cells need to be further studied.

## 5. Conclusions

Our study disclosed the immunologic modulations of SSW on responses of Th1 cells, Th17 cells, and Tregs in acute and chronic UC models. SSW might react on DSS-induced acute UC models by restraining the excessive immunoreaction of Th17 cells and facilitating the transdifferentiation from Th17 cells into IL-10^+^ Th17 cells, leading to the downregulation of IFN-*γ*^+^ Th17 cells/IL-10^+^ Th17 cells. SSW depressed the excessive reaction of Th1 cells and accelerated the development of Tregs, followed by enhancing the anti-inflammatory effects in chronic UC mice. This study implied that SSW can be an alternative prescription against UC via different immunologic regulations on the balance of Th1 cells, Th17 cells, and Tregs in acute and chronic UC, which might be correlated with the contents of cytokines, the frequencies of Th1 cells, Th17 cells, and Tregs, and the functional plasticity of Th17 cells.

## Figures and Tables

**Table 1:** The composition of SSW.

Official name	Chinese name	English name	Plant part	Usage (g)
*Myristica fragrans* Houtt.	Roudoukou	Myristicae semen	Kernel	200
*Psoralea corylifolia* L.	Buguzhi	Psoraleae fructus	Fruit	400
*Schisandra chinensis* Turcz. (Baill.)	Wuweizi	Schisandrae chinensis fructus	Fruit	200
*Euodia rutaecarpa* (Juss.) Benth.	Wuzhuyu	Euodiae fructus	Fruit	100
*Ziziphus jujube* Mill.	Dazao	Jujubae fructus	Fruit	200

## Data Availability

The data used to support the findings of the study are available from the corresponding author upon request.

## References

[1] Bressler B., Marshall J. K., Bernstein C. N. (2015). Clinical practice guidelines for the medical management of nonhospitalized ulcerative colitis: the toronto consensus. *Gastroenterology*.

[2] Zhu Q. Z., Zheng P., Chen X., Zhou F., He Q., Yang Y. (2018). Andrographolide presents therapeutic effect on ulcerative colitis through the inhibition of IL-23/IL-17 axis. *American Journal of Translational Research*.

[3] Farraye F. A., Odze R. D., Eaden J., Itzkowitz S. H. (2010). AGA technical review on the diagnosis and management of colorectal neoplasia in inflammatory bowel disease. *Gastroenterology*.

[4] Rogler G. (2014). Chronic ulcerative colitis and colorectal cancer. *Cancer Letters*.

[5] Gong W., Lv N., Wang B. (2012). Risk of ulcerative colitis-associated colorectal cancer in China: a multi-center retrospective study. *Digestive Diseases and Sciences*.

[6] Zhang L. G., Gao X., Zhou J. (2020). Increased risks of dental caries and periodontal disease in Chinese patients with inflammatory bowel disease. *International Dental Journal*.

[7] Hunter T. N., April N., Casey C., Amy L., Wendy K., yan D. (2019). Increasing trends in diagnostic prevalence and medication use among pediatric crohn’s disease patients in the United States. *Gastroenterology*.

[8] Chen F., Yin Y. T., Zhao H. M. (2020). Sishen pill treatment of DSS-induced colitis via regulating interaction with inflammatory dendritic cells and gut microbiota. *Frontiers in Physiology*.

[9] Hartmann F., Stein J., BudMesa-Study G. (2010). Clinical trial: controlled, open, randomized multicentre study comparing the effects of treatment on quality of life, safety and efficacy of budesonide or mesalazine enemas in active left-sided ulcerative colitis. *Alimentary Pharmacology & Therapeutics*.

[10] Kobayashi T., Fuse S., Sakamoto N. (2016). A new *Z* score curve of the coronary arterial internal diameter using the lambda-mu-sigma method in a pediatric population. *Journal of the American Society of Echocardiography*.

[11] Niu X., Fan T., Li W., Huang H., Zhang Y., Xing W. (2013). Protective effect of sanguinarine against acetic acid-induced ulcerative colitis in mice. *Toxicology and Applied Pharmacology*.

[12] Buffoni L., Piva M. M., Baska P. (2020). Immunization with the recombinant myosin regulatory light chain (FhrMRLC) in adjuplex(*R*) adjuvant elicits a Th1-biased immune response and a reduction of parasite burden in fasciola hepatica infected rats. *Parasitology International*.

[13] Bank S., Andersen P. S., Burisch J. (2018). Genetically determined high activity of IL-12 and IL-18 in ulcerative colitis and TLR5 in crohns disease were associated with non-response to anti-TNF therapy. *The Pharmacogenomics Journal*.

[14] Monteleone G. B., Biancone L., Marasco R. (1997). Interleukin 12 is expressed and actively released by crohn’s disease intestinal lamina propria mononuclear cells. *Gastroenterology*.

[15] Moschen A. R., Tilg H., Raine T. (2019). IL-12, IL-23 and IL-17 in IBD: immunobiology and therapeutic targeting. *Nature Reviews Gastroenterology & Hepatology*.

[16] Nielsen O. H., Kirman I., Rudiger N., Hendel J., Vainer B. Upregulation of interleukin-12 and -17 in active inflammatory bowel disease. *Scandinavian Journal of Gastroenterology*.

[17] Powell N., Walker A. W., Stolarczyk E. (2012). The transcription factor T-bet regulates intestinal inflammation mediated by interleukin-7 receptor+ innate lymphoid cells. *Immunity*.

[18] Zhu J., Yamane H., Paul W. E. (2010). Differentiation of effector CD4 T cell populations (∗). *Annual Review of Immunology*.

[19] Regmi S., Pathak S., Nepal M. R. (2019). Inflammation-triggered local drug release ameliorates colitis by inhibiting dendritic cell migration and Th1/Th17 differentiation. *Journal of Controlled Release*.

[20] Cipollini V., Anrather J., Orzi F., Iadecola C. (2019). Th17 and cognitive impairment: possible mechanisms of action. *Frontiers in Neuroanatomy*.

[21] Iwakura Y., Ishigame H. (2006). The IL-23/IL-17 axis in inflammation. *Journal of Clinical Investigation*.

[22] Acosta-Rodriguez E. V., Napolitani G., Lanzavecchia A., Sallusto F. (2007). Interleukins 1beta and 6 but not transforming growth factor-beta are essential for the differentiation of interleukin 17-producing human T helper cells. *Nature Immunology*.

[23] Ouyang W., Kolls J. K., Zheng Y. (1976). The biological functions of T helper 17 cell effector cytokines in inflammation. *Immunity*.

[24] Zheng Y., Danilenko D. M., Valdez P. (2007). Interleukin-22, a T(H)17 cytokine, mediates IL-23-induced dermal inflammation and acanthosis. *Nature*.

[25] Fernandez D., Flores-Santibanez F., Neira J. (2016). Purinergic signaling as a regulator of Th17 cell plasticity. *PLoS One*.

[26] Saraiva M., O’Garra A. (2010). The regulation of IL-10 production by immune cells. *Nature Reviews Immunology*.

[27] Zielinski C. E., Mele F., Aschenbrenner D. (2012). Pathogen-induced human TH17 cells produce IFN-gamma or IL-10 and are regulated by IL-1beta. *Nature*.

[28] Collison L. W., Workman C. J., Kuo T. T. (2007). The inhibitory cytokine IL-35 contributes to regulatory T-cell function. *Nature*.

[29] Niedbala W., Wei X. Q., Cai B. (2007). IL-35 is a novel cytokine with therapeutic effects against collagen-induced arthritis through the expansion of regulatory T cells and suppression of Th17 cells. *European Journal of Immunology*.

[30] Singh B., Read S., Asseman C. (2001). Control of intestinal inflammation by regulatory T cells. *Immunological Reviews*.

[31] Fantini M. C., Becker C., Tubbe I. (2006). Transforming growth factor beta induced FoxP3+ regulatory T cells suppress Th1 mediated experimental colitis. *Gut*.

[32] Commission C. P. (2015). *Pharmacopoeia of the People’s Republic of China-Volume I*.

[33] Chen H. (2011). The treatment of SSW on 30 cases of asdthenic splenonephro-yang. *Fujian University of Traditional Chinese Medicine*.

[34] Hu H. (2013). The efficacy of supplemented sishen wan combined with Chinese herbs retention enema on chronic ulcerative colitis. *CJTCM*.

[35] Xiaohua H. X. Z., Yi B. (2007). The efficacy of moxibustion and sishen wan on 32 cases of asdthenic splenonephro-yang. *Shaanxi Journal of Traditional Chinese Medicine*.

[36] Chengwei J. H. S. (2011). The effects of salicylazosulfapyridine combined with sishen wan granules retention enema on medium ulcerative colitis of asdthenic splenonephro-yang. *Tianjin University of Traditional Chinese Medicine*.

[37] Hu X. (2013). 36 cases of ulcerative colitis treated with sishen wan. *Chinese Medicine Modern Distance Education of China*.

[38] Wang Q. 26 cases of ulcerative colitis treated with Jiawei Sishen Wan. * J Mod Tradit Chin Med*.

[39] Ge W., Wang H. Y., Zhao H. M. (2020). Effect of sishen pill on memory T cells from experimental colitis induced by dextran sulfate sodium. *Frontiers in Pharmacology*.

[40] Wang H. Y., Zhao H. M., Wang Y. (2019). Sishen wan((R)) ameliorated trinitrobenzene-sulfonic-acid-induced chronic colitis via NEMO/NLK signaling pathway. *Frontiers in Pharmacology*.

[41] Zhao H. L., Duanyong, Tang F., Zuo Z. The protective mechanisms of sishen wan on the recovery of colonic mucosa in mice with ulcerative colitis. *Jounal Chin Trad Pat Med*.

[42] Wang Y. Z., Xiangdong, Duan Y., Li L., Cao Y., Wang B. (2014). Influence of the si shen pill on the histopathologic morphology, the balance between proinflammatory and anti-inflammatory cytokines in rats with ulcerative colitis. *Traditional Chinese Medicine Research*.

[43] Wang X. G., Dongyu, Li G., Qiu Z., Wu J., Hao Y. Comparison on effects of sishen pills and gegen qinlian tablets on DSS-induced experimental colitis in mice. *Chin J Exp Tradit Med Form*.

[44] Wang X. G., Dongyu, Qiu Z., Wu Y., Li G., Hao Y. Effects of sishen pill and gegen qinlian tablet on inflammatory cytokines in mice with colitis. *Chin J Inf Tradit Chin Med*.

[45] Cheung M. C., Spalding P. B., Gutierrez J. C. (2009). Body surface area prediction in normal, hypermuscular, and obese mice. *Journal of Surgical Research*.

[46] Ajayi B. O., Adedara I. A., Farombi E. O. (2018). Protective mechanisms of 6-gingerol in dextran sulfate sodium-induced chronic ulcerative colitis in mice. *Human & Experimental Toxicology*.

[47] Farombi E. O., Adedara I. A., Ajayi B. O., Idowu T. E., Eriomala O. O., Akinbote F. O. (2018). 6-gingerol improves testicular function in mice model of chronic ulcerative colitis. *Human & Experimental Toxicology*.

[48] Safaeian R., Howarth G. S., Lawrance I. C., Trinder D., Mashtoub S. (2019). Emu oil reduces disease severity in a mouse model of chronic ulcerative colitis. *Scandinavian Journal of Gastroenterology*.

[49] Hundorfean G., Neurath M. F., Mudter J. (2012). Functional relevance of T helper 17 (Th17) cells and the IL-17 cytokine family in inflammatory bowel disease. *Inflammatory Bowel Diseases*.

[50] Aggarwal A., Sabol T., Vaziri H. (2017). Update on the use of biologic therapy in ulcerative colitis. *Current Treatment Options in Gastroenterology*.

[51] Laroui H., Ingersoll S. A., Liu H. C. (2012). Dextran sodium sulfate (DSS) induces colitis in mice by forming nano-lipocomplexes with medium-chain-length fatty acids in the colon. *PLoS One*.

[52] Schmidt C., Dignass A., Hartmann F. (2011). [IBD ahead 2010—answering important questions in crohn’s disease treatment]. *Zeitschrift für Gastroenterologie*.

[53] Yin S., Yang H., Tao Y. (2020). Artesunate ameliorates DSS-induced ulcerative colitis by protecting intestinal barrier and inhibiting inflammatory response. *Inflammation*.

[54] Bauer C., Duewell P., Mayer C. (2010). Colitis induced in mice with dextran sulfate sodium (DSS) is mediated by the NLRP3 inflammasome. *Gut*.

[55] Liu Q., Zuo R., Wang K. (2020). Oroxindin inhibits macrophage NLRP3 inflammasome activation in DSS-induced ulcerative colitis in mice via suppressing TXNIP-dependent NF-kappaB pathway. *Acta Pharmacologica Sinica*.

[56] Valatas V., Bamias G., Kolios G. (2015). Experimental colitis models: insights into the pathogenesis of inflammatory bowel disease and translational issues. *European Journal of Pharmacology*.

[57] Gerlach K., Hwang Y., Nikolaev A. (2014). TH9 cells that express the transcription factor PU.1 drive T cell-mediated colitis via IL-9 receptor signaling in intestinal epithelial cells. *Nature Immunology*.

[58] Kaser A., Zeissig S., Blumberg R. S. (2010). Inflammatory bowel disease. *Annual Review of Immunology*.

[59] Schmitt H., Ulmschneider J., Billmeier U. (2020). The TLR9 agonist cobitolimod induces IL10-producing wound healing macrophages and regulatory T cells in ulcerative colitis. *Journal of Crohn’s and Colitis*.

[60] Ungaro R. M., Mehandru S., Allen P. B., Peyrin-Biroulet L., Colombel J. F. (2017). Ulcerative colitis. *Lancet*.

[61] Yang R. Y., Rongfeng (2013). 62 cases of ulcerative colitis treated with sishen wan and enema of herbal medicine. *Shaanxi Journal of Traditional Chinese Medicine*.

[62] Xie S. L., Jianjun Effectof four miraculous herbs decoction ultrafine particle in retention enema on 58 cases of ulcerative colitis with yang deficiency of spleen and kidney. *Chinese Medicine Modern Distance Education of China*.

[63] Zhao H. M., Liu Y., Huang X. Y. (2019). Pharmacological mechanism of Sishen Wan((R)) attenuated experimental chronic colitis by inhibiting wnt/beta-catenin pathway. *Journal of Ethnopharmacology*.

[64] Liu D. Y., Guan Y. M., Zhao H. M. (2012). The protective and healing effects of Si shen wan in trinitrobenzene sulphonic acid-induced colitis. *Journal of Ethnopharmacology*.

[65] Wang X. Y., Xueqin, Qiu Z., Ge D., Li G., Hao Y. Sishen pill ameliorates experimental colitis induced by trinitrobenzene sulfonic acid or dextran sulfate sodium in mice. *Journal of Beijing University of Traditional Chinese Medicine*.

[66] Chao K., Zhang S., Yao J. (2014). Imbalances of CD4(+) T-cell subgroups in crohn’s disease and their relationship with disease activity and prognosis. *Journal of Gastroenterology and Hepatology*.

[67] Han H. S., Shin J. S., Song Y. R. (2020). Immunostimulatory effects of polysaccharides isolated from young barley leaves (*Hordeum vulgare* L.) with dual activation of Th1 and Th2 in splenic T cells and cyclophosphamide-induced immunosuppressed mice. *International Journal of Biological Macromolecules*.

[68] Heller F., Florian P., Bojarski C. (2005). Interleukin-13 is the key effector Th2 cytokine in ulcerative colitis that affects epithelial tight junctions, apoptosis, and cell restitution. *Gastroenterology*.

[69] Neurath M. F., Fuss I., Schurmann G. (1998). Cytokine gene transcription by NF-kappa B family members in patients with inflammatory bowel disease. *Annals of the New York Academy of Sciences*.

[70] Bettelli E., Oukka M., Kuchroo V. K. (2007). T(H)-17 cells in the circle of immunity and autoimmunity. *Nature Immunology*.

[71] Dong C. (2008). TH17 cells in development: an updated view of their molecular identity and genetic programming. *Nature Reviews Immunology*.

[72] Gulen M. F., Bulek K., Xiao H. (2012). Inactivation of the enzyme GSK3alpha by the kinase IKKi promotes AKT-mTOR signaling pathway that mediates interleukin-1-induced Th17 cell maintenance. *Immunity*.

[73] Siakavellas S. I. B., Bamias G. (2012). Role of the IL-23/IL-17 axis in Crohn’s disease. *Discovery Medicine*.

[74] Li Q., Shan Q., Sang X., Zhu R., Chen X., Cao G. (2019). Total glycosides of peony protects against inflammatory bowel disease by regulating IL-23/IL-17 axis and Th17/treg balance. *The American Journal of Chinese Medicine*.

[75] Owaga E., Hsieh R. H., Mugendi B., Masuku S., Shih C. K., Chang J. S. (2015). Th17 cells as potential probiotic therapeutic targets in inflammatory bowel diseases. *International Journal of Molecular Sciences*.

[76] Palomino-Segura M., Latino I., Farsakoglu Y., Gonzalez S. F. (2020). Early production of IL-17A by gammadelta T cells in the trachea promotes viral clearance during influenza infection in mice. *European Journal of Immunology*.

[77] Zhang W., Xu L., Cho S. Y. (2016). Ginseng berry extract attenuates dextran sodium sulfate-induced acute and chronic colitis. *Nutrients*.

[78] Gao J., Cui J., Zhong H. (2020). Andrographolide sulfonate ameliorates chronic colitis induced by TNBS in mice via decreasing inflammation and fibrosis. *International Immunopharmacology*.

[79] Zhu J., Wang Y., Yang F. (2015). IL-33 alleviates DSS-induced chronic colitis in C57BL/6 mice colon lamina propria by suppressing Th17 cell response as well as Th1 cell response. *International Immunopharmacology*.

[80] Ueno A., Jeffery L., Kobayashi T., Hibi T., Ghosh S., Jijon H. (2018). Th17 plasticity and its relevance to inflammatory bowel disease. *Journal of Autoimmunity*.

[81] Mazzoni A., Maggi L., Siracusa F. (2019). Eomes controls the development of Th17-derived (non-classic) Th1 cells during chronic inflammation. *European Journal of Immunology*.

[82] Maggi L., Mazzoni A., Cimaz R., Liotta F., Annunziato F., Cosmi L. (2019). Th17 and Th1 lymphocytes in oligoarticular juvenile idiopathic arthritis. *Frontiers in Immunology*.

[83] Esplugues E., Huber S., Gagliani N. (2011). Control of TH17 cells occurs in the small intestine. *Nature*.

[84] Chistiakov D. A., Bobryshev Y. V., Kozarov E., Sobenin I. A., Orekhov A. N. (2014). Intestinal mucosal tolerance and impact of gut microbiota to mucosal tolerance. *Frontiers in Microbiology*.

[85] Eastaff-Leung N., Mabarrack N., Barbour A., Cummins A., Barry S. (2010). Foxp3+ regulatory T cells, Th17 effector cells, and cytokine environment in inflammatory bowel disease. *Journal of Clinical Immunology*.

[86] Gorelik L., Flavell R. A. (2002). Transforming growth factor-beta in T-cell biology. *Nature Reviews Immunology*.

[87] Lu Y., Xu Y., Zhang S. (2019). Human gingiva-derived mesenchymal stem cells alleviate inflammatory bowel disease via IL-10 signalling-dependent modulation of immune cells. *Scandinavian Journal of Immunology*.

[88] Wirtz S., Billmeier U., McHedlidze T., Blumberg R. S., Neurath M. F. (2011). Interleukin-35 mediates mucosal immune responses that protect against T-cell-dependent colitis. *Gastroenterology*.

[89] Rosen M. J., Karns R., Vallance J. E. (2017). Mucosal expression of type 2 and type 17 immune response genes distinguishes ulcerative colitis from colon-only crohn’s disease in treatment-naive pediatric patients. *Gastroenterology*.

[90] Wang Z. Y., Sato H., Kusam S., Sehra S., Toney L. M., Dent A. L. (2005). Regulation of IL-10 gene expression in Th2 cells by jun proteins. *The Journal of Immunology*.

[91] Lohneis P., Wienert S., Klauschen F., Anagnostopoulos I., Johrens K. (2017). Fibrosis in low-grade follicular lymphoma—a link to the TH2 immune reaction. *Leukemia and Lymphoma*.

[92] Suzuki M., Yokota M., Nakamura Y., Ozaki S., Murakami S. (2016). Intranasal administration of IL-35 inhibits allergic responses and symptoms in mice with allergic rhinitis. *Allergology International*.

